# Tianeptine: An Antidepressant with Memory-Protective Properties

**DOI:** 10.2174/157015908787386096

**Published:** 2008-12

**Authors:** Phillip R Zoladz, Collin R Park, Carmen Muñoz, Monika Fleshner, David M Diamond

**Affiliations:** 1 Medical Research Service, VA Hospital, Tampa, Florida, USA; 2 Department of Psychology, University of South Florida, Tampa, Florida, USA; 3 Departments of Molecular Pharmacology & Physiology, University of South Florida, Tampa, Florida, USA; 4 Center for Preclinical and Clinical Research on PTSD, University of South Florida, Tampa, Florida, USA; 5 Institut de Recherches Internationales Servier, Courbevoie, France; 6 Center for Neuroscience and Department of Integrative Physiology, University of Colorado, Boulder, Colorado, USA

**Keywords:** Depression, tianeptine, stress, memory, synaptic plasticity, animal models.

## Abstract

The development of effective pharmacotherapy for major depression is important because it is such a widespread and debilitating mental disorder. Here, we have reviewed preclinical and clinical studies on tianeptine, an atypical antidepressant which ameliorates the adverse effects of stress on brain and memory. In animal studies, tianeptine has been shown to prevent stress-induced morphological sequelae in the hippocampus and amygdala, as well as to prevent stress from impairing synaptic plasticity in the prefrontal cortex and hippocampus. Tianeptine also has memory-protective characteristics, as it blocks the adverse effects of stress on hippocampus-dependent learning and memory. We have further extended the findings on stress, memory and tianeptine here with two novel observations: 1) stress impairs spatial memory in adrenalectomized (ADX), thereby corticosterone-depleted, rats; and 2) the stress-induced impairment of memory in ADX rats is blocked by tianeptine. These findings are consistent with previous research which indicates that tianeptine produces anti-stress and memory-protective properties without altering the response of the hypothalamic-pituitary-adrenal axis to stress. We conclude with a discussion of findings which indicate that tianeptine accomplishes its anti-stress effects by normalizing stress-induced increases in glutamate in the hippocampus and amygdala. This finding is potentially relevant to recent research which indicates that abnormalities in glutamatergic neurotransmission are involved in the pathogenesis of depression. Ultimately, tianeptine’s prevention of depression-induced sequelae in the brain is likely to be a primary factor in its effectiveness as a pharmacological treatment for depression.

## INTRODUCTION

Depression is a widespread, recurrent mental disorder that has detrimental effects on individuals, as well as society, at large [44,189]. Although considerable progress has been made in characterizing the neurobiological sequelae that result from this disorder, the factors that are responsible for depression’s development and progression are not well understood. Research indicates that there is a heritable component to depression, and, more recently, investigators have identified candidate genes that appear to increase one’s susceptibility for the disorder [98,184]. This area of research has provided insight into the etiology of depression with the finding that gene polymorphisms interact with environmental factors, such as stressful events, to increase the likelihood that a person will develop major depression [15,17,62,82, 112,206].

For the past few decades, the prevailing view has been that depression results from abnormally low levels of monoamine neurotransmitter substances (e.g., serotonin, norepinephrine, dopamine), which is commonly known as the monoamine hypothesis [12,22,171]. Support for this hypothesis was based on the incidental finding that efficacious antidepressants, such as monoamine oxidase inhibitors and tricyclics, increased monoamine neurotransmitter levels [127]. Therefore, the primary focus of pharmacotherapy for depression has been to prescribe agents which are known to increase levels of the neurotransmitter serotonin, and today, selective serotonin reuptake inhibitors (SSRIs), such as fluoxetine, paroxetine and sertraline, are the most prescribed pharmacological treatments for this disorder [8,195].

Recent research suggests that increasing the levels of monoamines provides only an indirect contribution to antidepressant actions [186]. Moreover, some findings are inconsistent with the monoamine hypothesis of depression, thereby suggesting that the neurochemical basis of the disorder is more complicated than previously considered. For instance, traditional antidepressants ameliorate depressive symptoms only in a subset of patients, despite their low levels of monoamines [12], and are largely ineffective for people with severe forms of depression [113,137]. In these latter cases, clinicians may resort to electroconvulsive shock therapy (ECT), which has proven to be one of the most effective treatments for severe, pharmacologically-resistant forms of depression [136,147]. Despite its effectiveness, however, ECT’s mechanism of action remains largely unknown. 

An alternative and well-established treatment for depression is tianeptine, an antidepressant which does not share pharmacological properties with TCAs, MAOIs or SSRIs [12,18,95,145,191]. Early studies suggested that tianeptine enhanced the uptake of serotonin [28,78,124,200], but more recent work indicates that tianeptine’s actions as an antidepressant are independent of modulating serotonin levels [145,193]. Instead, tianeptine’s primary mode of action is to influence the expression of synaptic plasticity [18,36,55, 77,120,121] *via* the modulation of glutamatergic neurotransmission [88,118,158,159]. Tianeptine’s effectiveness in treating depression is of clinical, as well as conceptual, significance. That is, the contrast in mechanistic actions between SSRIs and tianeptine, combined with the observation that both types of agents can treat depression, serves as a challenge to the heuristic value of the monoamine hypothesis of depression [66,135].

## CHRONIC STRESS AND STRUCTURAL PLASTICITY IN THE HIPPOCAMPUS, PREFRONTAL CORTEX AND AMYGDALA

In recent years, researchers have suggested that depression is manifested through alterations in neuroplasticity, which involves structural and functional changes in how the brain processes information [55,77,191]. Investigators have contended that the emotional and cognitive components of depression manifest themselves as changes in neurochemical levels that ultimately produce significant alterations in brain morphology and, consequently, function [55]. In depressed patients, studies have described structural and functional alterations in three brain regions that are highly involved in emotional and cognitive processing: the hippocampus, prefrontal cortex and amygdala [174].

In general, studies have reported significant reductions of hippocampal and prefrontal cortex volumes in depressed patients [74,111,194]. The hippocampus is a medial temporal lobe structure which is important for declarative memory in humans [49,182] and spatial working memory in rodents [20,21,79,128,129,207]. The prefrontal cortex is located in the anterior portion of the frontal lobe and plays an important role in complex cognitive processes, such as planning, decision-making and behavioral flexibility [13]. Depressed individuals exhibit impaired performance on hippocampus- and prefrontal cortex-dependent cognitive tasks, which corresponds with reduced or abnormal activity in each of these brain regions when depressed patients engage in such tasks [47,126]. In contrast to the hippocampus and prefrontal cortex, amygdala volumes of depressed patients are larger than those of healthy individuals following the first episode of depression [54]. However, with recurrent episodes, amygdala volumes in depressed patients tend to be smaller than those of controls [175]. Nevertheless, most work has reported that activity of the amygdala is increased in depressed individuals [43] and with successful treatment, significantly declines [178].

It is well-established that stress significantly increases one’s likelihood of developing depression [81,132]. Extensive preclinical research has shown that chronic stress produces behavioral alterations that are analogous to those observed in depressed patients (e.g., anhedonia, learned helplessness, cognitive impairments) [4,56,114]. Thus, researchers have utilized animal models of stress effects on brain and behavior to potentially develop a better understanding of the neurobiological sequelae of this disorder. Animal models have shown that chronic stress significantly reduces the length, spine density and arborization of dendrites on neurons located in the prefrontal cortex [26,33,99,152,153] and hippocampus [31,87,97,106,108,122,198,203], while increasing each one of these parameters on neurons in the amygdala [197,198]. Not surprisingly, then, these chronic stress regimens have been shown to produce significant impairments of hippocampus-dependent (e.g., spatial learning) [14,92,104,139,181,185,211] and prefrontal cortex-dependent (e.g., attention set-shifting, reversal learning) memory [26,99], while enhancing performance on tasks that are dependent upon the amygdala (e.g., fear conditioning) [32, 167]. Additionally, the same chronic stress that leads to hypertrophy of cells in the amygdala increases the expression of anxiety-like behaviors in rats tested in the elevated plus maze [197,198].

It is important to note that the effects of chronic stress on hippocampal [31,181] and prefrontal cortex [151] morphology have been found to be reversible – that is, the dendrites re-grew when the stress was discontinued. This was not the case, however, for the effects of chronic stress on amygdala morphology or the amygdala-mediated expression of anxiety-like behavior [199]. Additional work showed that the effects of chronic stress on these brain regions were mediated by an interaction between glucocorticoids and NMDA receptor activity. Thus, chronic administration of corticosterone mimicked the effects of chronic stress on hippocampal [109,181,208] and prefrontal cortex morphology [204], and the stress-induced dendritic retraction observed in the hippocampus was blocked by steroid synthesis inhibitors [107], as well as NMDA receptor antagonists [107] and agents that significantly reduced extracellular levels of glutamate (e.g., phenytoin) [108,201]. These findings resonate with research in depressed patients, which indicates that these individuals have an overactive HPA axis [57,138] and abnormal brain glutamatergic levels [80,94,166].

## TIANEPTINE PREVENTS CHRONIC STRESS-INDUCED STRUCTURAL AND FUNCTIONAL CHANGES IN THE HIPPOCAMPUS, PREFRONTAL CORTEX AND AMYGDALA

Daily administration of tianeptine blocks the chronic stress-induced reduction of hippocampal volume [34], as well as the retraction of CA3 dendrites in the hippocampus [31,105,202]. In contrast, the SSRIs fluoxetine and fluvoxamine were ineffective in preventing the stress-induced changes in CA3 morphology [105], suggesting that the effects of tianeptine and SSRIs may be mediated, at least in part, by different cellular and molecular mechanisms. Additional work has shown that tianeptine also prevents the effects of chronic stress on hippocampus-dependent learning and memory [30,104,212]. Investigators have yet to determine whether or not tianeptine prevents the effects of chronic stress on prefrontal cortex morphology. Considering tianeptine’s ability to block the effects of chronic stress on hippocampal structure and function, it is likely that tianeptine would exert positive effects on the prefrontal cortex, as well. Tianeptine also blocks the effects of chronic stress on hypertrophy of amygdala dendritic arbors, as well as the concurrent enhancement of anxiety-like behavior accompanying chronic stress [144]. These findings may be relevant to other work reporting that chronic tianeptine treatment reduced the expression of auditory fear conditioning, an amygdala-dependent task [23].

## MECHANISMS UNDERLYING TIANEPTINE’S EFFECTS ON CHRONIC STRESS-INDUCED CHANGES IN BRAIN STRUCTURE AND FUNCTION

The hippocampus is one of only two brain regions in the adult mammalian brain that produces new neurons, a process known as neurogenesis [48]. Although the functional role of neurogenesis has remained a highly debated topic, studies have provided evidence linking hippocampal neurogenesis with hippocampus-dependent learning [59,177]. In addition, several researchers have hypothesized that the pathogenesis of depression involves impaired hippocampal neurogenesis [42,46,65,69,169]. Accordingly, in animal models, chronic stress significantly reduces hippocampal neurogenesis [60, 63,64,143,165,172] and increases apoptotic cell death in the hippocampus and temporal cortex [63,103,210]. Clinically effective antidepressants, including tianeptine, prevent the effects of chronic stress on hippocampal neurogenesis [34, 42,172]. Tianeptine has also been reported to block the chronic stress-induced increase in apoptotic cell death in the temporal cortex [102], which may be related to its prevention of the chronic stress-induced reduction of cerebral metabolites associated with neuronal viability (e.g., *N*-acetyl-aspartate) [34].

Neurotrophic factors are significant regulators of cell survival and proliferation, thus making them vitally important for the process of neurogenesis [68]. Some of the most extensively characterized neurotrophic factors include nerve growth factor (NGF), brain-derived neurotrophic factor (BDNF), neurotrophin-3 (NT-3) and neurotrophin-4 (NT-4). Numerous studies have shown that acute and chronic stress significantly reduce neurotrophic factor levels [172,179,180, 190], but most studies have focused on the stress-induced reduction of BDNF levels in the hippocampus [3,10,90,131, 146,155,161,162,165,170]. This effect has become the center of attention, at least in part, because several studies have reported significantly reduced levels of serum and hippocampal BDNF in depressed patients [6,75,176]. BDNF knock-out mice have been reported to exhibit morphological changes in the hippocampus that are comparable to those observed following exposure to chronic restraint stress [157]. Interestingly, investigators have shown that the efficacy of antidepressants in ameliorating behavioral symptoms of depression, in depressed patients and animal models of stress, depends on their ability to increase BDNF levels [6,25,29].

Tianeptine’s ability to prevent the effects of chronic stress on neurogenesis may involve blocking the stress-induced reduction of neurotrophic factor levels in the hippocampus [3]. Another study, although reporting no effect of stress or tianeptine on hippocampal BDNF, found that chronic tianeptine treatment significantly increased BDNF levels in the rat amygdala, independent of whether or not the rats were exposed to stress [157]. According to Reagan and colleagues, the amygdala may be the site of initiation of chronic stress-induced morphological changes in other brain regions, such as the hippocampus and prefrontal cortex [157,159]. In support of this hypothesis, clinical studies on depressed patients have reported that morphological changes in the amygdala precede those that are observed in the hippocampus [117]. Therefore, tianeptine’s effectiveness as antidepressant treatment may result from its stabilization of neurotrophin levels in the amygdala. Future studies should be conducted to examine this hypothesis.

## STRESS, TIANEPTINE AND SYNAPTIC PLASTICITY IN THE HIPPOCAMPUS, PREFRONTAL CORTEX AND AMYGDALA

Researchers have also hypothesized that depression involves a disruption of long-term synaptic plasticity [36,121]. To indirectly address this issue, investigators have examined the effects of stress on long-term potentiation (LTP), a physiological model of learning and memory involving an enhancement of synaptic efficacy following high-frequency stimulation of afferent fibers [156]. Extensive work has shown that stress impairs the induction of LTP in the hippocampus and prefrontal cortex, while facilitating its induction in the amygdala [37,39,41,85]. The stress-induced modulation of synaptic plasticity has been shown to be mediated by interactions among glucocorticoids [89,110,115], glutamatergic NMDA receptors [84,116,148] and amygdala-induced modulation of hippocampal plasticity [1,2].

Tianeptine has been shown to block the stress-induced impairment of LTP in the hippocampus and prefrontal cortex, without interfering with the stress-induced enhancement of LTP in the basolateral amygdala (BLA) [72,164,173,196]. Tianeptine blocked the inhibitory effects of stress on hippocampal LTP and primed burst potentiation (PBP), a low-threshold form of LTP, when it was administered before or after the stress experience [173,196]. Other antidepressants, including some SSRIs, have also been reported to block the effects of stress on LTP in the hippocampus and prefrontal cortex, although these effects have been less significant and more transitory in nature [164]. 

## MEMORY-PROTECTIVE EFFECTS OF TIANEPTINE

Tianeptine administration, under non-stress conditions, has been shown to increase the magnitude of synaptic plasticity (LTP and PB potentiation) in the hippocampal CA1 region [173,196]. This finding suggests that tianeptine should enhance learning and memory. Indeed, studies have shown that tianeptine enhances spontaneous alternation behavior, as well as performance on discrimination tasks in the T-maze and radial arm maze [70,123]. In contrast, the SSRI fluoxetine *impaired* performance on the radial arm maze discrimination task [70], a finding that is relevant to other work reporting that fluoxetine impairs the induction of LTP in hippocampal slices [173]. Recent work from our laboratory has shown that the acute administration of tianeptine immediately before training in the radial-arm water maze (RAWM) enhanced long-term (24 hr) spatial memory [130]. The doses of tianeptine used in this experiment (1-10 mg/kg) are the same doses that have been shown to enhance hippocampal LTP and PB potentiation [173,196], and to block the effects of chronic stress on hippocampal morphology and hippocampus-dependent learning and memory.

Extensive research has shown that acute stress impairs hippocampus-dependent learning and memory in humans and rodents [37,39,41,83,85]. We recently reported that tianeptine, but not the anxiolytic propranolol, blocked the predator stress-induced impairment of rat spatial memory in the RAWM [24]. Tianeptine prevented the effects of stress on memory without altering the stress-induced increase in glucocorticoids, which suggests that tianeptine’s memory-protective effects are independent of the stress-induced activation of the HPA axis. Moreover, the findings are consistent with *in vivo *electrophysiological studies reporting that tianeptine blocked the effects of stress on hippocampal LTP without affecting stress-induced increases in corticosterone levels in rats [173].

To extend this observation, we have tested whether tianeptine could prevent the stress-induced impairment of spatial memory in adrenalectomized (ADX) rats. If tianeptine’s mechanism of action is independent of the stress-induced increase in adrenal hormones, then tianeptine should prevent the effects of stress on hippocampus-dependent spatial memory in ADX rats. In this experiment, rats (250-275 g; Harlan Laboratories; Indianapolis, IN) underwent ADX or sham surgery, following previously-described methods [52,133]. The drinking water of ADX rats was composed of 0.9% saline with 25 mg/l of corticosterone to prevent any adverse effects of corticosterone depletion on their physiology. One week following surgery, the rats were injected intraperitoneally with tianeptine (10 mg/kg) or vehicle (0.9% saline, 1 ml/kg) and then, 30 min later, they were given 12 trials to learn the location of a hidden escape platform, which was placed at the end of one of six arms, in the RAWM [24, 38,140,142,211]. Arm entry errors (i.e., entries into arms that did not contain the hidden platform) served as an indicator of a rat’s memory for the hidden platform. Following training, the rats spent a 30 min delay period in their home cages (No Stress) or confined to a small plexiglas chamber near a cat (Stress) [24,142,168]. The 30 min delay period was terminated with a single memory test trial in the RAWM, which was followed by the collection of a 0.5 ml sample of tail blood for subsequent analysis of corticosterone levels [24, 142,168].

As can be seen in the memory test trial data on the left side of Fig. (**1**), the control (i.e., non-stressed) rats demonstrated excellent memory for the location of the hidden platform, independent of whether or not they had undergone ADX surgery. This finding indicates that adrenal hormones are not necessary for spatial learning in the water maze and successful retrieval of short-term (30 min) hippocampus-dependent memory. As shown on the right side of Fig. (**1**), water maze training increased serum corticosterone levels in sham-operated control rats, relative to control rats that had undergone the ADX procedure. We found that vehicle-treated, adrenal-intact and ADX rats that were exposed to the cat during the 30 min delay period displayed significantly impaired performance on the memory test trial. This finding supports the notion that increased levels of glucocorticoids do not underlie the rapid impairing effects of stress on hippocampus-dependent memory [140]. Most importantly, acute administration of tianeptine prior to water maze training prevented the stress-induced impairment of spatial memory in both ADX and sham-operated animals, without having any significant effect on serum corticosterone levels.

In conjunction with our prior work [24], these findings provide convincing evidence that tianeptine’s memory-protective effects are not accomplished *via* the modulation of stress-induced increases in glucocorticoid levels. This series of electrophysiological and behavioral experiments supports the hypothesis that tianeptine enables hippocampus-dependent information to be stored more efficiently, thereby protecting its retrieval from being disrupted by stress.

## MECHANISMS UNDERLYING TIANEPTINE’S ANTIDEPRESSANT AND MEMORY-PROTECTIVE PROPERTIES

Although it initially appeared that tianeptine’s antidepressant action was attributable to its effects on serotonin reuptake [78,96], recent work indicates that its therapeutic effects may be more associated with its modulation of the glutamatergic system [18,77]. Glutamate is the primary excitatory neurotransmitter of the central nervous system, and one of its roles is to regulate calcium influx by acting on postsynaptic AMPA and NMDA receptors [160]. Studies have shown that depressed patients exhibit elevated glutamate levels in plasma, CSF and post-mortem brain samples, which supports current views implicating the dysregulation of glutamate transmission in the pathogenesis of depression [80,94,166]. 

Extensive work has implicated hyperactivity of the glutamatergic system in the deleterious effects of stress on brain structure and function. Experiments conducted primarily on the hippocampus have shown that stress significantly increases glutamate levels [7,100,101,125,159], inhibits glutamate uptake [209], increases the expression and binding of glutamate receptors [11,93,119] and increases calcium currents [73]. Accordingly, researchers have shown that administration of NMDA receptor antagonists blocks the effects of stress on behavioral, morphological and electrophysiological measures of hippocampal function [84,107,141].

Tianeptine appears to protect the hippocampus and prefrontal cortex from the deleterious effects of stress by normalizing the stress-induced modulation of glutamatergic activity. For instance, tianeptine blocked the stress-induced increase in NMDA channel currents, as well as the ratio of NMDA:non-NMDA receptor currents, in the CA3 region of the hippocampus [88]. Tianeptine also inhibited the acute stress-induced increase in extracellular levels of glutamate in the basolateral amygdala (BLA), while having no effect on the stress-induced increase in these levels in the central nuclei of the amygdala (CeA) [159]. Interestingly, as mentioned above, tianeptine had no effect on the stress-induced enhancement of LTP in the BLA [196]. This finding suggests that the stress-induced enhancement of LTP in the BLA may involve NMDA-independent forms of synaptic plasticity, such as voltage-gated calcium channel-dependent LTP [91]. 

In contrast to tianeptine, administration of the SSRI fluoxetine increased baseline and stress-induced levels of glutamate in the BLA and CeA [159]. This finding may explain why SSRIs are anxiogenic early in the treatment phase and exert therapeutic antidepressant and anxiolytic effects only after a substantial delay [23,58]. Moreover, investigators have shown that acute administration of the SSRI citalopram enhanced the acquisition of auditory fear conditioning, while chronic treatment with citalopram impaired the acquisition and expression of conditioned fear [23]. Acute treatment with tianeptine, in contrast, had no effect on auditory fear conditioning, but when given chronically, exerted effects comparable to those of citalopram. Thus, tianeptine demonstrates long-lasting anxiolytic and antidepressant effects that are similar to SSRIs, without the adverse acute effects typically found with these agents.

Tianeptine’s effect on glutamatergic activity in amygdala may play an important role in its ability to reverse the effects of chronic stress on amygdala morphology and the expression of anxiety-like behaviors. In addition to its glutamatergic modulation, tianeptine reduces the expression of corticotropin-releasing hormone (CRH) mRNA in the amygdala and the bed nucleus of the stria terminalis (BNST), a brain region that is highly innervated by amygdala fibers [86]. CRH neurotransmission in both of these regions has been implicated in the expression of anxiety-like behaviors, and several studies have reported significantly elevated CSF CRH levels in depressed patients [67,76,183]. If the amygdala is the site of the initiation of chronic stress-induced functional changes in other brain regions, such as the hippocampus and prefrontal cortex, then tianeptine’s ability to stabilize amygdala activity could underlie its widespread anti-stress effects.

Chronic stress has been shown to increase expression of the glutamate transporter, GLT-1, which is important for removing excess glutamate from synaptic regions [158]. This effect was specifically observed in the CA3 region of the hippocampus, the primary area exhibiting significant morphological alterations following chronic stress. Researchers have postulated that the up-regulation of GLT-1 levels in this region is a compensatory response to chronic elevations of extracellular glutamate levels. Importantly, tianeptine has been shown to block the stress-induced increase in hippocampal GLT-1 levels. In theory, tianeptine accomplishes this feat by normalizing stress-induced glutamate levels in the hippocampus, thereby removing the stimulus (i.e., excessive glutamate) which necessitates increased expression of GLT-1.

Despite its ability to normalize the stress-induced increase in NMDA receptor currents, tianeptine also increases basal excitatory synaptic transmission in hippocampal circuits, predominantly *via* enhancing AMPA EPSCs [88]. In addition to NMDA receptors, AMPA receptors play an important role in excitatory synaptic transmission and the induction of long-term synaptic plasticity [154]. Recent work has reported that tianeptine modulates the phosphorylation of AMPA receptor subunits in the hippocampus [186]. Other antidepressants, such as SSRIs and tricyclics, have been shown to increase phosphorylation of the Ser845 site on the glutamate receptor subunit 1 (GluR1) of hippocampal AMPA receptors [45,187]. Investigators found that chronic, but not acute, tianeptine treatment significantly increased phosphorylation of the Ser831 and Ser845 sites on the GluR1 of AMPA receptors in the CA3 region of the hippocampus [186]. Typically, phosphorylation of the Ser831 and Ser845 sites of AMPA receptors occurs *via* protein kinase A (PKA) and calcium/calmodulin-dependent protein kinase II (CaMKII) or protein kinase C (PKC), respectively, and potentiates AMPA currents in the hippocampus [9,163]. Thus, the tianeptine-mediated increase in the phosphorylation of the serine sites on the GluR1 of AMPA receptors could explain the finding of a tianeptine-induced enhancement of AMPA EPSCs in the study of Kole *et al*. [88], which may also be relevant toward understanding tianeptine’s effectiveness as an antidepressant.

Recent work has also reported that tianeptine has anticonvulsant properties. Uzbay and colleagues found that tianeptine reduced the intensity [27] and delayed the onset [192] of pentylenetetrazole-induced seizures in rodents. The latter effect was blocked by the administration of caffeine, a nonspecific adenosine receptor antagonist, and 8-cyclopentyl-1,3-dipropylxanthine, an A_1_ receptor-specific antagonist. However, administration of the A_2_ receptor-specific antagonist, 8-(3-chlorostyryl) caffeine, had no effect on the tianeptine-induced delay of seizure onset, suggesting that tianeptine’s anticonvulsant properties are dependent upon activation of A_1_ adenosine receptors. Since previous work has shown that activation of A_1_ adenosine receptors has anxiolytic effects [53,71,149,150], this specific category of adenosinergic receptors could be responsible, at least in part, for tianeptine’s anxiolytic effects in rodents [23,50,51,144] and in the depressed population [35,205].

## SUMMARY AND CONCLUSIONS

Depression is a common mental disorder for which effective pharmacological treatments are lacking. Investigators have utilized animal models of depression to develop a better understanding of the neurobiological basis of this disorder, which could ultimately produce improved treatment options for the patient. We have reviewed the findings of preclinical research demonstrating that tianeptine prevents the deleterious effects of stress on physiology and behavior. Tianeptine prevents chronic stress-induced morphological changes in the hippocampus and amygdala and blocks the effects of acute stress on synaptic plasticity in the hippocampus and prefrontal cortex. We have also reviewed findings demonstrating that tianeptine has memory-protective properties, in which tianeptine-treated rats exhibited intact hippocampus-dependent memory despite their being exposed to powerful fear-provoking stressors. Tianeptine’s prevention of the adverse effects of stress on brain and behavior is likely to contribute to its effectiveness as a treatment for people suffering from major depressive disorder. Although the antidepressant effects of tianeptine in people have been obtained through chronic administration, studies on the acute effects of tianeptine provide researchers with important information regarding tianeptine’s mechanism of action and ways in which its use may be expanded in humans.

Tianeptine’s actions do not appear to involve the modulation of stress-induced changes in HPA activity. We previously reported that tianeptine blocked the stress-induced impairment of spatial memory without affecting the stress-induced increase in glucocorticoid levels. Here, we have found that tianeptine prevented the acute stress-induced impairment of spatial memory in adrenalectomized rats, thereby demonstrating conclusively that elevated levels of glucocorticoids are not necessary for acute stress to affect memory, nor are they involved in tianeptine’s protective actions on memory. More recent work has suggested that tianeptine’s antidepressant effects may be attributable to its normalization of the stress-induced alterations of glutamatergic neurotransmission [18,77]. This finding resonates with accumulating evidence that has implicated abnormal glutamate activity in the pathogenesis of depression. Other research has shown that tianeptine has anticonvulsant properties, which are dependent upon adenosine receptor activation. Given the involvement of adenosine receptors in anxiolytic effects on behavior, tianeptine’s antidepressant effects could also involve modulation of adenosinergic neurotransmitter systems.

In summary, tianeptine is a well-described antidepressant with effective actions against stress-induced sequelae of the nervous system. It is as effective as SSRIs in treating depression, produces fewer adverse side effects and reduces anxious symptoms associated with depression without the need for concomitant anxiolytic therapy [5,16,19,61,188]. It is therefore relevant to note that tianeptine ameliorates symptoms in people with post-traumatic stress disorder (PTSD) [134] and in recent work has been shown to block the effects of intense stress on behavior and cardiovascular systems in an animal model of PTSD [212]. Thus, the well-described antidepressant and memory protective properties of tianeptine indicate that, in addition to its effectiveness as a treatment in mood disorders, it potentially has broader applications, as in the treatment of anxiety.

## Figures and Tables

**Fig. (1):**
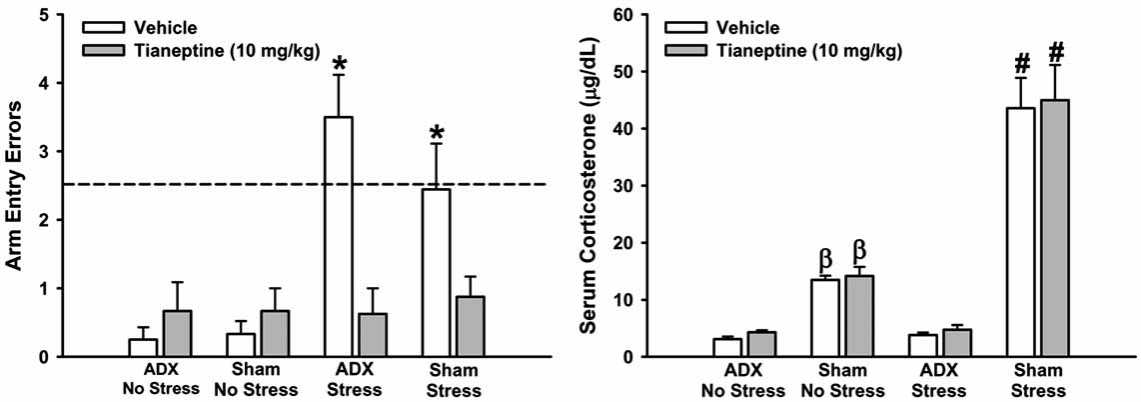
Pre-training administration of tianeptine blocked the effects of predator stress on spatial memory in adrenalectomized (ADX) and adrenal-intact (Sham) rats without affecting corticosterone levels. Arm entry errors from the 12 acquisition trials in the RAWM (data not shown) were analyzed with a mixed-model ANOVA. This analysis revealed significant main effects of trials, *F*(11,693) = 19.59, and drug, *F*(1,63) = 4.28, and a significant Surgery x Stress x Drug interaction, *F*(1,63) = 4.18 (*p*’s < 0.05). While all groups made significantly fewer arm entry errors as trials progressed, tianeptine led to significantly more arm entry errors than vehicle in all groups except for the stress-exposed ADX group. All other main effects and interactions were not significant. Arm entry errors from the 30 min memory test trial (left) were analyzed with a one-way ANOVA. This analysis revealed significant main effects of stress, *F*(1,63) = 20.64, and drug, *F*(1,63) = 9.22, as well as a significant Stress x Drug interaction, *F*(1,63) = 18.22 (*p*’s < 0.01). Vehicle-treated rats exposed to predator stress during the 30 min delay period made significantly more arm entry errors than control (i.e., unstressed) rats. The administration of tianeptine prior to training blocked this effect in both ADX and sham-operated animals. Serum corticosterone levels (right) were analyzed with a one-way ANOVA. This analysis revealed significant main effects of surgery, *F*(1,41) = 129.71, and stress, *F*(1,41) = 49.75, and a significant Surgery x Stress interaction, *F*(1,41) = 46.08 (*p*’s < 0.0001). Water maze training significantly increased corticosterone levels in sham-operated control rats, relative to ADX controls. Predator stress significantly increased corticosterone levels in sham-operated, but not ADX, rats, an effect that was independent of tianeptine treatment. For the water maze data, the dashed line at 2.5 errors indicates chance level of performance [40]. * = *p* < 0.05 relative to no stress groups and tianeptine-treated stress groups; β = *p* < 0.05 relative to stress groups and ADX-no stress groups; # = *p* < 0.05 relative to no stress groups and ADX-stress groups.
